# A Theoretical Study and Numerical Simulation of a Quasi-Distributed Sensor Based on the Low-Finesse Fabry-Perot Interferometer: Frequency-Division Multiplexing

**DOI:** 10.3390/s17040859

**Published:** 2017-04-14

**Authors:** José Trinidad Guillen Bonilla, Alex Guillen Bonilla, Verónica M. Rodríguez Betancourtt, Héctor Guillen Bonilla, Antonio Casillas Zamora

**Affiliations:** 1 Computing and Electronic Departments, Centro Universitario de Ciencias Exactas e Ingenierías (CUCEI), University of Guadalajara, Blvd. M. García Barragán 1421, Guadalajara 44410, Jalisco, Mexico; 2 Mathematic Department, Centro Universitario de Ciencias Exactas e Ingenierías (CUCEI), University of Guadalajara, Blvd. M. García Barragán 1421, Guadalajara 44410, Jalisco, Mexico; 3 Departamento de Ciencias Computacionales, Centro Universitario de los Valles, Universidad de Guadalajara, Ameca Km 45.5, Ameca C.P. 46600, Jalisco, Mexico; 4 Materials Science Graduate School, Centro Universitario de Ciencias Exactas e Ingenierías (CUCEI), University of Guadalajara, Blvd. M. GarcíaBarragán 1421, Guadalajara 44410, Jalisco, Mexico

**Keywords:** quasi-distributed sensor, low-finesse Fabry-Perot interferometer, sensor simulation, frequency-domain multiplexing and resolution vs. signal-to-noise ratio

## Abstract

The application of the sensor optical fibers in the areas of scientific instrumentation and industrial instrumentation is very attractive due to its numerous advantages. In the industry of civil engineering for example, quasi-distributed sensors made with optical fiber are used for reliable strain and temperature measurements. Here, a quasi-distributed sensor in the frequency domain is discussed. The sensor consists of a series of low-finesse Fabry-Perot interferometers where each Fabry-Perot interferometer acts as a local sensor. Fabry-Perot interferometers are formed by pairs of identical low reflective Bragg gratings imprinted in a single mode fiber. All interferometer sensors have different cavity length, provoking frequency-domain multiplexing. The optical signal represents the superposition of all interference patterns which can be decomposed using the Fourier transform. The frequency spectrum was analyzed and sensor’s properties were defined. Following that, a quasi-distributed sensor was numerically simulated. Our sensor simulation considers sensor properties, signal processing, noise system, and instrumentation. The numerical results show the behavior of resolution vs. signal-to-noise ratio. From our results, the Fabry-Perot sensor has high resolution and low resolution. Both resolutions are conceivable because the Fourier Domain Phase Analysis (FDPA) algorithm elaborates two evaluations of Bragg wavelength shift.

## 1. Introduction

Bragg grating has a very particular peak in its reflection spectrum; the peak is centered at the Bragg wavelength $\lambda_{BG}=2n\Lambda$ [1], where Λ is the grating pitch and n is the effective fiber refraction index. The operational principle of a fiber Brag grating sensor is based on the spectral shift of the central Bragg wavelength due to the variation of the pitch and refraction index because of temperature or strain change on the grating. The monitoring system needs to detect the wavelength shift with very high resolution, permitting its correct evaluation. This shift is evaluated from optical measurements, for example, a dual-OFC FBG (OFC: Optical Frequency Combs and FBG: Fiber Bragg Grating) interrogation system [2], tunable Fabry-Perot filter with a piezoelectric actuator [3], and direct spectroscopic detection [4]. 

Bragg gratings play an important role in fiber-optic sensor technology. Such sensors are very attractive for quasi-distributed sensing, employing only one optical fiber with many gratings printed along a fiber length. The conventional Bragg grating sensors use a broadband light source and a direct spectrometric detection technique. Their principal problem concerns the detection of relatively small shifts in the resonant wavelength of grating arrays exposed to strain or slow temperature changes. An additional application of Bragg gratings in sensor technology is to build interferometers within a single path fiber. In this case, Bragg gratings act as selective mirrors. The positions of gratings along the fiber length define the optical path difference. Frequency-division multiplexing, wavelength-division multiplexing, and time-division multiplexing can be implemented [5,6,7,8,9,10,11,12].

The low-finesse Fabry-Perot interferometer has low reflectivity and it can be built with the end-faces of the lead-in/out and the target fibers [13], polymer sensing film [14], chirped Bragg gratings [15], Si plates and single mode optical fibers [16], the fiber end and a mirror [17], micro bubble structure [18], dielectric thin films [19] and Bragg gratings [20,21,22]. In particular, the low-finesse Fabry-Perot interferometer based on Bragg gratings has important advantages, for example, high accuracy, excellent measurement sensitivity, potential industrial application, easy implementation, easy multiplexing, security, and the electromagnetic field does not affect the interferometry sensor. Whereas, its disadvantages are its fragility and high cost. To reduce the cost per sensing point, the capability of the multiplexing topology and the multiplexing technique was increased. By applying frequency-division multiplexing, wavelength-division multiplexing, and wavelength-frequency-division multiplexing techniques, the quasi-distributed fiber optic sensors can be developed. These quasi-distributed sensors can measure temperature [23,24], strain [25], and vibration [26]. 

The twin-grating fiber optic sensor was used for the temperature measurement. The optical sensor acts as a low-finesse Fabry-Perot interferometer and it consists of two identical Bragg gratings separated by a short distance. The Fourier Domain Phase Analysis (FDPA) algorithm was used for its signal demodulation. The FDPA algorithm evaluates the Bragg wavelength shift at the frequency domain. The algorithm is based on the evaluation of the phase of the interference pattern produced by light reflected from both gratings and on the determination of the Bragg wavelength shift. The wavelength shift sensitivity was measured to 0.00985 nm/°C [27]. This fiber sensor was also used for the measurement of static strain. Resolution of 0.2 µm/m was reported [28].

In reference [29], a quasi-distributed sensor was experimentally proposed. Twin-grating sensors were applied as local sensors. Frequency-division multiplexing was implemented. In reference [30], this quasi-distributed sensor was described. The authors described the application of frequency-division multiplexing. A tunable external cavity diode laser was used for the sensor interrogation. The sensing systems consisted of a serial array of 14 twin grating sensors. All Bragg gratings had the same length of 0.5 mm and reflectivity of 0.8%. The Bragg wavelength of all gratings was 1550.6 nm. The cavities were into the interval of 2 mm to 34 mm. The optical spectrum was acquired. Their frequency components were separated applying the fast Fourier transform (FFT) algorithm. There were 14 channels. Each channel was generated from each Fabry-Perot sensor. Other quasi-distributed fiber optic sensors can be found in references [31,32,33,34,35,36].

To our knowledge, the quasi-distributed sensor described in [30] does not have an analytic analysis. Therefore, local sensor limitations are not known. Here, a theoretical analysis and numerical simulation is elaborated for the quasi-distributed sensor described in reference [30]. A broadband light source, direct spectrometric detection technique, and frequency-domain multiplexing are considered in our study. Knowing its operation principle, the optical spectrum was represented mathematically. We analyzed the optical signal and then the quasi-distributed sensor’s properties were defined, for example, minimum and maximum cavities, number of samples, spatial resolution, and multiplexing capability of a twin-grating fiber sensor. All parameters are expressed in terms of physical parameters and instrumentation characteristics. Then, the quasi-distributed sensor was numerically simulated (in operation) and we obtained the graph of demodulation errors vs. signal-to-noise ratio. From our numerical results, the cavity length augments the resolution and all Fabry-Perot sensors have two resolutions: a high resolution and low resolution. The cavity length, low resolution, and noise system define the transition between both resolutions. In general, our theoretical analysis and numerical simulation permit its optimal implementation and its design. 

## 2. Optical Signal

**Figure 1:**
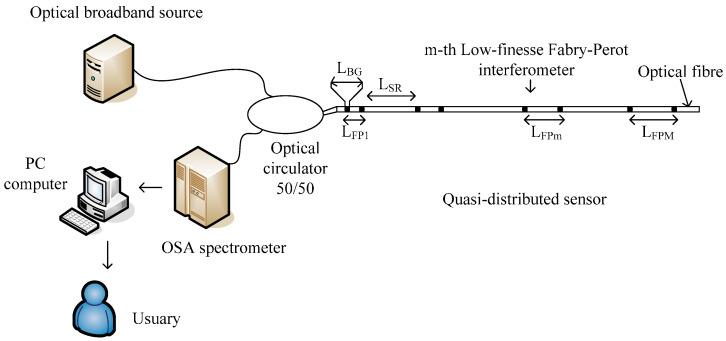
Sensing system: $L_{BG}$ is the length of gratings, $L_{FP1}$ is the minimum cavity length, $L_{SR}$ is the spatial resolution, $L_{FPm}$ is the *m*-th cavity length, $L_{FPM}$ is the maximum cavity length and OSA is the Optical Spectrometer Analyzer.

Figure 1 shows our optical sensing system schematically. The optical system consists of a broadband source, an optical circulator 50/50, an optical spectrometer analyzer (OSA spectrometer), a personal computer and a quasi-distributed sensor. The quasi-distributed sensor can be implemented by using a serial array of low-finesse Fabry-Perot interferometers [29,30]. The local sensors are formed by pairs of identical low reflective Bragg gratings imprinted in a single mode fiber. Each Fabry-Perot interferometer has a unique optical path length which obtains the frequency-division multiplexing (FDM). The Bragg gratings have approximately the same length and typical reflectivity of 0.1%. Thus, wavelength-division multiplexing was eliminated for our optical sensor.

### 2.1. $R_{T}\left(\lambda \right)$ and $R_{T}\left(\nu \right)$ Spectrums

When the quasi-distributed sensor does not have external perturbations, the optical signal $R_{T}\left(\lambda \right)$ will be the superposition of all interference patterns,

(1) $$R_{T}\left(\lambda \right)=\overset{M}{\underset{m=1}{\sum }}R_{m}\left(\lambda \right)=R_{1}\left(\lambda \right)+R_{2}\left(\lambda \right)+R_{3}\left(\lambda \right)+\ldots +R_{M}\left(\lambda \right)$$

$R_{T}\left(\lambda \right)$ is the optical signal detected by the OSA spectrometer and $R_{1}\left(\lambda \right),R_{2}\left(\lambda \right),R_{3}\left(\lambda \right)\ldots R_{M}\left(\lambda \right)$ are interference patterns generated by all interferometer sensors. Considering the physical parameters, the optical signal can be re-written as [27]

(2) $$R_{T}\left(\nu \right)=\overset{M}{\underset{m=1}{\sum }}2a_{m}\left[{\left(\frac{\pi n_{1}L_{BG}}{\lambda_{BG}}\right)}^{2}\sin\;c^{2}\left(\frac{2n_{1}L_{BG}(\lambda -\lambda_{BG})}{\lambda_{BG}^{2}}\right)\right]\left[1+\cos\left(\frac{4\pi nL_{FPm}(\lambda -\lambda_{BG})}{\lambda_{BG}^{2}}\right)\right]$$

where λ is the wavelength, $a_{m}$ is amplitude factor, $n_{1}$ is the amplitude of the effective refractive index modulation of the gratings, $L_{BG}$ is the length of gratings, $\lambda_{BG}$ is the Bragg wavelength, n is the effective index of the core, $L_{FPm}$ is the mth cavity length, and M is the number of low-finesse Fabry-Perot interferometers (local sensors). Analyzing the optical signal (2), all interference patterns have a similar enveloped function (sinc function), the sinc function is the reflection spectrum of the gratings, the width $\Delta_{BG}$ is defined as the spectral distance between its +1 and −1 zeros,

(3) $$\Delta_{BG}=\frac{\lambda_{BG}^{2}}{n_{1}L_{BG}}$$

Each interference pattern has its own frequency component. There are M modulate functions where the frequency component $\nu_{FPm}$ will be

(4) $$\nu_{FPm}=\frac{2nL_{FPm}}{\lambda_{BG}^{2}}$$

To know the frequency components, we apply the Fourier transform to the optical signal

(5) $$R_{T}\left(\nu \right)=ℱ\left\{R_{T}\left(\lambda \right)\right\}=\overset{\infty }{\underset{-\infty }{\int }}R_{T}\left(\lambda \right)e^{-i2\pi \nu \lambda }d\lambda$$

$R_{T}\left(\nu \right)$ is the frequency spectrum, ℱ{ } is the Fourier operator, and ν is the frequency. Substituting Equations (2)–(4) into Equation (5), the frequency spectrum is

(6) $$R_{T}\left(\nu \right)=\overset{\infty }{\underset{-\infty }{\int }}\overset{M}{\underset{m=1}{\sum }}2a_{m}\left[{\left(\frac{\pi n_{1}L_{BG}}{\lambda_{BG}}\right)}^{2}\sin\;c^{2}\left(\frac{\lambda -\lambda_{BG}}{\Delta_{BG}}\right)\right]\left[1+\cos\left(2\pi \nu_{FPm}\left(\lambda -\lambda_{BG}\right)\right)\right]e^{-i2\pi \nu \lambda }d\lambda$$

Invoking the convolution properties and Fourier operator, we have

(7) $$R_{T}\left(\nu \right)=ℱ\left\{\left[{\left(\frac{\pi n_{1}L_{BG}}{\lambda_{BG}}\right)}^{2}\sin\;c^{2}\left(\frac{\lambda -\lambda_{BG}}{\Delta_{BG}}\right)\right]\right\}⊗ℱ\left\{\overset{M}{\underset{m=1}{\sum }}2a_{m}\left[1+\cos\left(2\pi \nu_{FPm}\left(\lambda -\lambda_{BG}\right)\right)\right]\right\}$$

the symbol ⊗ indicates the convolution. Using the identities $\cos^{2}\left(\varphi \right)=\frac{1}{2}\left(1+\cos\left(2\varphi \right)\right)$ , $\cos\left(\varphi \right)=\frac{e^{i\varphi }+e^{-i\varphi }}{2}$ , $\sum_{m=1}^{M}e^{-i\varphi_{m}}=\sum_{m=-M}^{-1}e^{i\varphi_{m}}$ , then solving the frequency spectrum R(ν) is

(8) $$R_{T}\left(\nu \right)=\overset{M}{\underset{m=-M}{\sum }}R_{m}\left(\nu \right)=\overset{M}{\underset{m=-M}{\sum }}c_{m}tri\left(\frac{\nu -\nu_{FPm}}{\nu_{BG}}\right)$$

$R_{T}\left(\nu \right)$ spectrum is a set of triangle functions where the function tri(x) is defined as $tri\left(x\right)=\{\begin{array}{cc} 1-\left|x\right| & \left|x\right|\leq 1\\ 0 & otherwise \end{array}$ , $c_{m}$ are amplitude factors, and $\nu_{BG}$ is the bandwidth

(9) $$\nu_{BG}=\frac{4n_{1}L_{BG}}{\lambda_{BG}^{2}}$$

**Figure 2:**
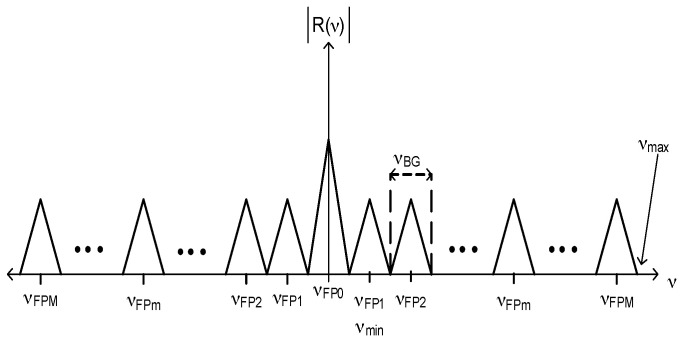
R(ν) frequency spectrum.

In addition, $\nu_{FPm}$ is the center position of each triangle function. Here, all frequency components were separated as Figure 2 illustrates.

### 2.2. $R_{T}\left(\lambda ,\delta \lambda \right)$ and $R_{T}\left(\nu ,\delta \lambda \right)$ Spectrums

When the quasi-distributed sensor has external perturbations, the measured temperature or string affects the gating period Λ , the refraction index n , the length of gratings $L_{BG}$ , and cavity length $L_{FPm}$ [27]. In turn, interference patterns have a small shift in response to a measured variation, and the optical signal detected by the OSA spectrometer is

(10) $$R_{T}\left(\lambda ,\delta \lambda \right)=\overset{M}{\underset{m=1}{\sum }}2a_{m}\left[{\left(\frac{\pi n_{1}L_{BG}}{\lambda_{BG}}\right)}^{2}sinc^{2}\left(\frac{2n_{1}L_{BG}(\lambda -\lambda_{BG}-\delta \lambda_{BGm})}{\lambda_{BG}^{2}}\right)\right]\left[1+\cos\left(\frac{4\pi nL_{FPm}(\lambda -\lambda_{BG}-\delta \lambda_{BGm})}{\lambda_{BG}^{2}}\right)\right]$$

The optical spectrum $R_{T}\left(\lambda ,\delta \lambda \right)$ can be expressed as

(11) $$R_{T}\left(\lambda ,\delta \lambda \right)=\overset{M}{\underset{m=1}{\sum }}R_{m}\left(\lambda -\delta \lambda_{BGm}\right)=R_{1}\left(\lambda -\delta \lambda_{BG1}\right)+R_{2}\left(\lambda -\delta \lambda_{BG2}\right)+\ldots +R_{M}\left(\lambda -\delta \lambda_{BGM}\right)$$

where $R_{T}\left(\lambda ,\delta \lambda \right)$ is the optical signal due to external perturbations and $\delta \lambda_{BGm}$ is the Bragg wavelength shift due to measured change. Now, we estimate their frequency components through

(12) $$R_{T}\left(\nu ,\delta \lambda \right)=ℱ\left\{R_{T}\left(\lambda ,\delta \lambda \right)\right\}=\overset{\infty }{\underset{-\infty }{\int }}\overset{M}{\underset{m=1}{\sum }}R_{m}\left(\lambda -\delta \lambda_{BGm}\right)e^{-i2\pi \nu \lambda }d\lambda$$

Invoking the shift property, the Fourier transform is

(13) $$R_{T}\left(\nu ,\delta \lambda \right)=\overset{M}{\underset{m=-M}{\sum }}R_{m}\left(\nu \right)e^{-i2\pi \nu \delta \lambda_{BGm}}$$

Observing the Equation (13), the frequency spectrum $R_{T}\left(\nu ,\delta \lambda \right)$ is the multiplication between $R_{T}\left(\nu \right)$ (Equation (8)) and a set of phases. Those phases contain the information about the perturbations.

## 3. Cavity Length

For all quasi-distributed sensors based on interferometers (optical fiber), the cavity length is a very important parameter since it defines the sensor characteristics. Their limits depend of instrumentation, local sensor characteristics, and signal demodulation. In the following sections, we determine minimum and maximum cavities where the low-finesse Fabry-Perot interferometer can be applied. 

### 3.1. Minimum Cavity Length

The Fourier Domain Phase Analysis (FDPA) algorithm was developed for the twin-grating fiber optic sensor [27]. This algorithm does not accept additional information and does not lose information, therefore, good signal detection and good frequency component identification are necessary. From Figure 2, first frequency components $\nu_{FP1}$ can be defined by

(14) $$\nu_{FP1}=\nu_{BG}$$

The condition (14) eliminates the overlapping between components, $\nu_{FP1}$ and $\nu_{FP0}$ . Using the Equations (4) and (9), we have

(15) $$\frac{2nL_{FP1}}{\lambda_{BG}^{2}}=\frac{4n_{1}L_{BG}}{\lambda_{BG}^{2}}$$

As $n_{1}\approx n$ , the minimum cavity length will be

(16) $$L_{FP1}=2L_{BG}$$

It is not possible to have smaller cavities because the FDPA algorithm cannot demodulate the optical signal.

### 3.2. Maximum Cavity Length

The optical sensing system applies the direct spectroscopic detection [4]. This technique uses an optical spectrometer analyzer which defines the maximum cavity length $L_{FPM}$ . The OSA spectrometer has a limit for the optical signal detection. The limit is the Full-With Half-Maximum (FWHM). Considering the sampling theorem, the OSA spectrometer can detect the signal if and only if the next condition is true,

(17) $${\Delta \lambda }_{\mathrm{FPmin}}=2\Delta \lambda$$

where ${\Delta \lambda }_{\mathrm{FPmin}}$ is the minimum period detectable (FWHM) and Δλ is its spectrometer resolution. Then, the maximum frequency component can be expressed as

(18) $$\nu_{FPM}=\frac{1}{{\Delta \lambda }_{\mathrm{FPmin}}}$$

From Figure 2 and Equation (4), last frequency component $\nu_{FPM}$ can be determined by

(19) $$\nu_{FPM}=\frac{2nL_{FPM}}{\lambda_{BG}^{2}}$$

Combining Equations (17)–(19), the maximum cavity length is

(20) $$L_{FPM}=\frac{\lambda_{BG}^{2}}{4n\Delta \lambda }$$

Equation (20) indicates the maximum cavity length where OSA spectrometer can detect the optical signal. It is not possible to have bigger cavities because the instrumentation cannot detect the optical signal. Using Equations (16) and (20), the cavity length can be within the interval of

(21) $$2L_{BG}\leq L_{FP}\leq \frac{\lambda_{BG}^{2}}{4n\Delta \lambda }$$

## 4. Capacity of Frequency-Division Multiplexing

In the quasi-distributed sensor, each low-finesse Fabry-Perot interferometer generates an interference pattern and then each pattern produces a channel in the frequency domain. The enveloped function produces the bandwidth $\nu_{BG}$ and the modulate function provokes the frequency components $-\nu_{FPm}$ , $\nu_{FP0},$ and $\nu_{FPm}$ . The term $\nu_{FP0}$ contains information from all Fabry-Perot interferometers while $-\nu_{FPm}$ and $\nu_{FPm}$ contain similar information from the *mth* Fabry-Perot sensor. From Figure 2, we have the next condition

(22) $$M=\frac{\nu_{FPM}}{\nu_{FP1}}$$

In other words, the capacity of frequency-division multiplexing M is given by the relation between last and first frequency components. Substituting the Equations (14), (15), and (17) into (22), the capacity M can be re-written as

(23) $$M=\frac{L_{FPM}}{L_{FP1}}$$

Finally, substituting the Equations (16) and (20) into Equation (22), we have

(24) $$M=\frac{\lambda_{BG}^{2}}{8nL_{BG}\Delta \lambda }$$

This expression gives the limit for the multiplexing capacity within one wavelength channel.

## 5. Number of Samples

When the optical spectrometer analyzer instrument acquires the optical signal, the reflection spectrum is recorded as a series of digital samples. If a minimum and maximum wavelength within a working interval $\lambda_{w}=\lambda_{max}-\lambda_{min}$ , then $\lambda_{max}$ is the maximum wavelength, $\lambda_{min}$ is the minimum wavelength and δλ is the wavelength step. The signal samples $R_{T}\left(\lambda_{k}\right)$ are taken as wavelengths $\lambda_{k}=\lambda_{min}+k\delta \lambda$ where k=0, 1, 2,…,N−1 , N is the number of samples. The representation of such a signal in the Fourier domain is also discrete. Therefore, we obtain the next condition from Figure 2

(25) $$\nu_{s}\geq 2\nu_{max}=2\left(\nu_{FPM}+\frac{\nu_{BG}}{2}\right)$$

where $\nu_{max}$ is the maximum frequency, $\nu_{s}$ is the sampling frequency, and the Nyquist theorem was considered. Substituting Equations (9) and (19) into Equation (25), we have

(26) $$\nu_{s}\geq \frac{4n}{\lambda_{BG}^{2}}\left(L_{FPM}+2L_{BG}\right)$$

Since $\nu_{s}=\frac{1}{\delta \lambda }$ , we have

(27) $$\delta \lambda \leq \frac{\lambda_{BG}^{2}}{4n\left(L_{FPM}+2L_{BG}\right)}$$

Finally, the number of samples is

(28) $$N=\frac{\lambda_{w}}{\delta \lambda }=\frac{4\lambda_{w}n\left(L_{FPM}+2L_{BG}\right)}{\lambda_{BG}^{2}}$$

The number of samples depends of optical system parameters.

## 6. Digital Demodulation

The demodulation is the complete signal processing algorithm developed for a quasi-distributed sensor based on the low-finesse Fabry-Perot interferometers. The complete processing algorithm combines the Fourier Domain Phase Analysis (FDPA) algorithm and a bank of *M* filters. The FDPA algorithm was described in [27] while the bank of filters is

(29) $$F\left(\nu \right)=rect\left(\frac{\nu }{\nu_{BG}}\right)⊗\overset{M}{\underset{m=1}{\sum }}\delta \left(\nu -\nu_{FPm}\right)$$

where the symbol ⊗ indicates the convolution operation, the rect function is definition as

(30) $$\mathrm{rect}\left(\nu \right)=\{\begin{array}{cc} 1 & \left|\nu \right|<\frac{\nu_{BG}}{2}\\ 0 & \left|\nu \right|>\frac{\nu_{BG}}{2} \end{array}$$

where δ is the Dirac delta. Invoking the Dirac delta properties, the bank of *M* filters is

(31) $$F\left(\nu \right)=\overset{M}{\underset{m=1}{\sum }}rect\left(\frac{\nu -\nu_{FPm}}{\nu_{BG}}\right)$$

The bank filter of *M* filters is a series of rect functions where $\nu_{FPm}$ is the central position and $\nu_{BG}$ is its bandwidth.

The digital demodulation consists of two phases: calibration and measurements. In the calibration, there are four steps: (1) $R_{T}\left(\lambda \right)$ is acquired, (2) $R_{T}\left(\nu \right)$ is computed, (3) $R_{m}\left(\nu \right)$ is filtered $R_{m}\left(\nu \right)=R_{T}\left(\nu \right)F\left(\nu \right),$ and (4) we calculate its complex conjugate $R_{m}^{*}\left(\nu \right)$ where * indicates a complex conjugate. In the measurement, there are seven steps: (1) $R_{T}\left(\lambda ,\delta \lambda \right)$ is acquired, (2) $R_{T}\left(\nu ,\delta \lambda \right)$ is computed, (3) ${\tilde{R}}_{m}\left(\nu ,\delta \lambda \right)$ is filtered ${\tilde{R}}_{m}\left(\nu ,\delta \lambda \right)=R_{T}\left(\nu ,\delta \lambda \right)F\left(\nu \right)$ , (4) the relative phase $\varphi_{rel}$ is calculated, (5) the ambiguity 2πP is eliminated and then absolute phase $\varphi_{abs}$ is calculated, (6), the Bragg wavelength shift is computed, and (7) a digital adaptive filter is applied [37].

**Figure 3:**
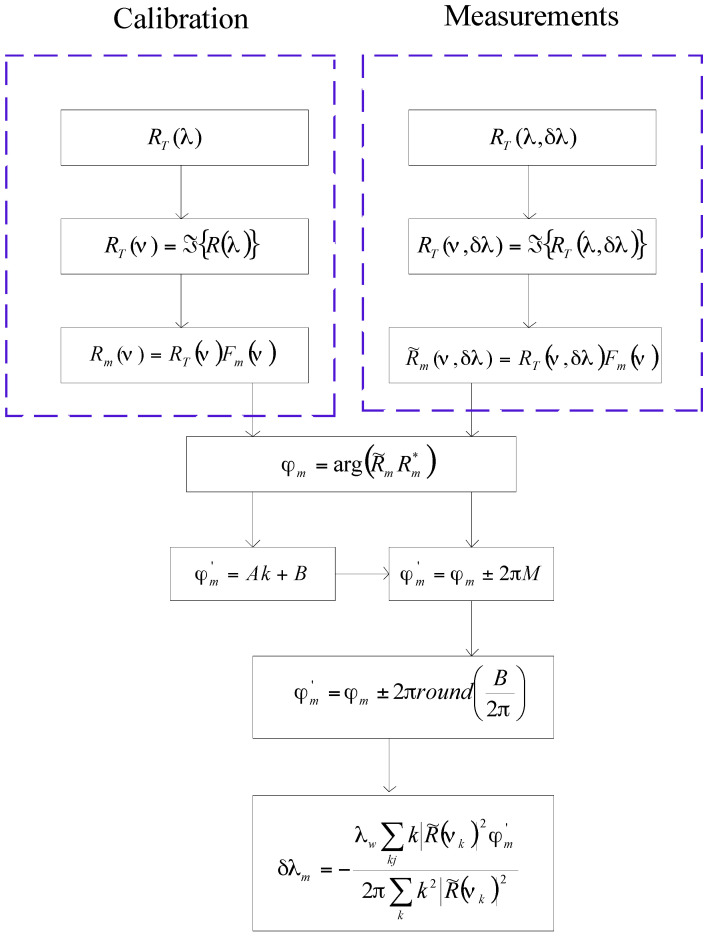
Digital demodulation represented schematically.

Due to the presence of the noise in the original signal, the calculated phase will be fluctuating. To minimize the noise influence and provide the best estimate, the absolute phase is multiplied with a set of coefficients. Those coefficients act as an adaptive filter. Figure 3 illustrates the digital demodulation schematically.

## 7. Numerical Simulation and Discussion

### 7.1. Parameters and Results

**Figure 4:**
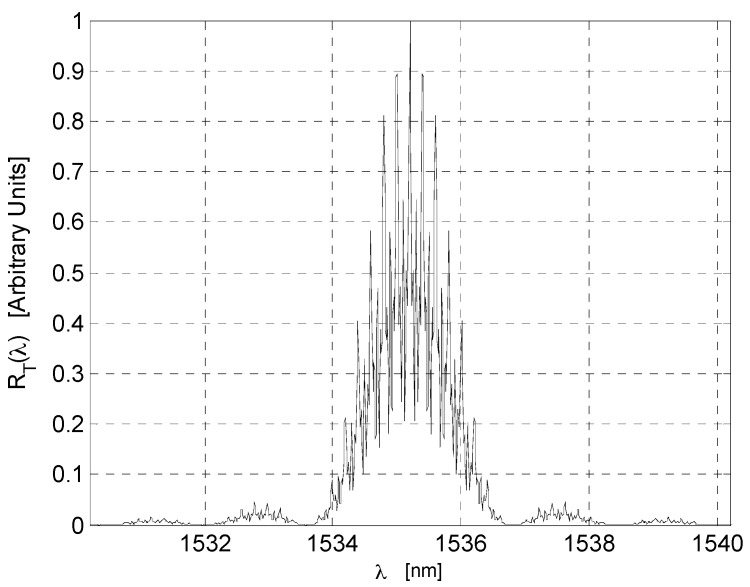
Optical signal $R_{T}\left(\lambda \right)$ .

**Figure 5:**
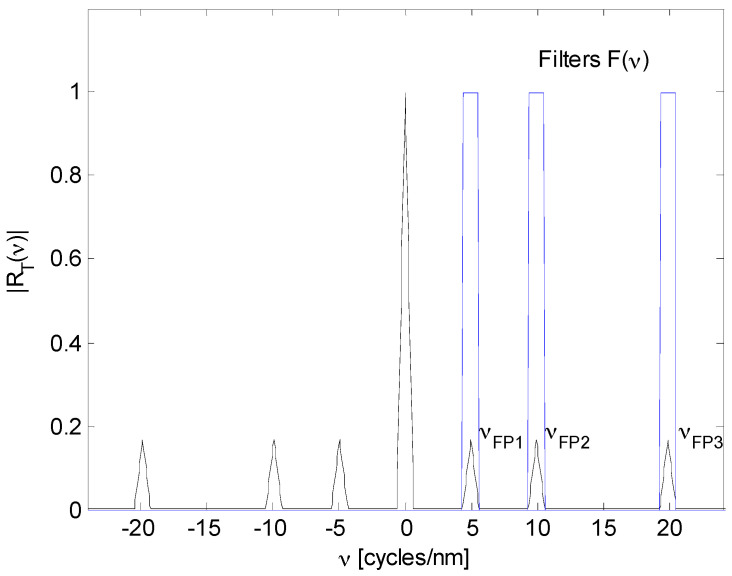
Optical signal $R_{T}\left(\nu \right)$ .

**Figure 6:**
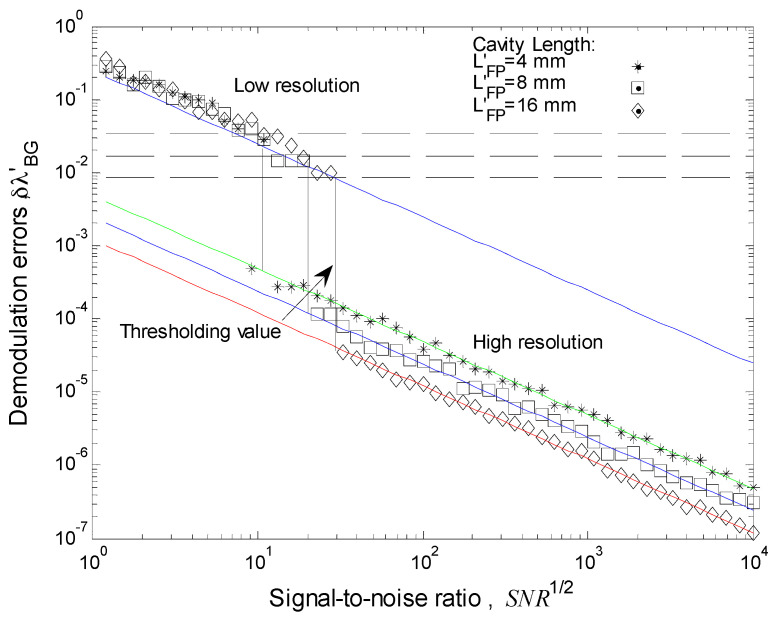
Numerical results.

To test and compare our theoretical analysis, we performed a numerical simulation of a quasi-distributed sensor based on low-finesse Fabry-Perot interferometers. Three Fabry-Perot sensors were simulated. Their physical parameters can be observed in Table 1. Discrete spectrums were simulated using the physical parameters. Noise was simulated by adding to those samples pseudorandom numbers with Gaussian distribution; the interval was from $\sqrt{SNR}=10^{0}$ to $\sqrt{SNR}=10^{4}$ . Typical of Bragg gratings with rectangular profiles, a refractive index modulation was used. In most of our numerical experiments, the number of samples was equal to 1024 (Fast Fourier transform algorithm was considered). For each local sensor, the reference spectrum and 50 measurements were simulated. The measurements were in the intervals of S1 → 0 to 0.2 nm, S2 → 0 to 0.4 nm, and S3 → 0 to 0.7nm. Figure 4 shows the spectrum $R_{T}\left(\lambda \right)$ , Figure 5 shows the spectrum $R_{T}\left(\nu \right),$ and Figure 6 presents our numerical results: Demodulation errors vs SNR^1/2^. A Laptop Toshiba 45C was used, with 512 Mb of RAM memory and a velocity of 1.7 GHz.

If the OSA spectrometer has Δλ=10 pm (typical value), the quasi-distributed sensor will have its limits as Table 2 illustrates.

From Table 1 and Table 2, the simulated quasi-distributed sensor satisfies the instrumentation and signal requirements. Observing Table 1 and Figure 4 and Figure 5, numerical results are in concordance with the theory. Thus, we confirm our theoretical analysis. Our numerical results can be observed in Figure 6. The theoretical analysis and our numerical results are in concordance with experimental results presented by Shlyagin et al. [30]; frequency-division multiplexing can be implemented based on a twin grating sensor. The presented study optimizes significantly the quasi-distributed sensor implementation and the sensibility of local sensors. To develop the sensing system based on the frequency-division multiplexing (Figure 1), the broadband light source can have the following parameters: a central wavelength of $\lambda_{c}=1532.5\;\mathrm{nm}$ , $\lambda_{min}=1520\;\mathrm{nm}$ , and $\lambda_{max}=1570\;\mathrm{nm}$ . The low reflectivity eliminates the cross-talk noise and its value can be selected from the references [29,38].

Figure 6 shows the behavior of Demodulation errors vs signal-to-noise rate SNR^1/2^. If the demodulation error is denominated resolution, then low-finesse Fabry-Perot has two resolutions: low resolution and high resolution. Two resolutions are possible because the FDPA algorithm does two evaluations of the Bragg wavelength shift [27,37]. All Fabry-Perot sensors have similar low resolution, however, each local sensor has its own high resolution. The high resolution depends of cavity length. If the cavity length is bigger than the Fabry-Perot sensor, it will have better resolution. 

### 7.2. Discussion

Based on our theoretical analysis and numerical simulation, the quasi-distributed sensor would be built on the low-finesse Fabry-Perot interferometer. Our theoretical analysis optimizes its implementation. Instrumentation, local sensor properties, noise (Gaussian distribution), and signal processing were considered. The quasi-distributed sensor has good sensitivity and excellent resolution. All Fabry-Perot sensors have two resolutions: low resolution and high resolution (See Figure 6). Low resolution was obtained when the Bragg wavelength shift was evaluated with an enveloped function. High resolution was obtained when the Bragg wavelength shift was evaluated combining the enveloped and modulated functions [27,37]. 

When the noise is big, signal-to-noise ratio (SNR) is small. In this case, the FDPA algorithm cannot evaluate the Bragg wavelength shift, causing the transition from high resolution to low resolution. This can be observed in Figure 6. As the (necessary) signal is within the interval of –π to π , and based on the signal detection theory, the thresholding value is

(32) $$3\sigma_{env}<\frac{\Delta \lambda_{FPm}}{2}$$

where $\sigma_{env}$ is the low resolution (resolution by enveloped function) and $\Delta \lambda_{FPm}=\frac{1}{\nu_{FPm}}$ is the period of our frequency component. The threshold divides between low and high resolutions. Substituting Equation (4) into Equation (32), we have

(33) $$\sigma_{env}<\frac{\lambda_{BG}^{2}}{12nL_{FPm}}$$

From Equation (33), each low-finesse Fabry-Perot interferometer has its own thresholding value. This one depends on the cavity length, Bragg wavelength, and refraction index. For example: our Fabry-Perot sensors have next thresholding values, S1 → 0.033 nm, S2 → 0.016 nm, and S3 → 0.008 nm. The thresholding value is smaller if the cavity length is bigger. 

In the quasi-distributed sensor, ghost interferometers are eliminated if the separation between any two interferometers satisfies the expression $L_{sp}>L_{FPM}$ , where $L_{sp}$ is the spatial resolution. If Fabry-Perot interferometers are formed by uniform unapodized gratings with equal length $L_{BG}$ , the bandwidth of each peak is given by Equation (9). To be separated in the frequency domain, two peaks should not overlap. This condition imposes the following constraints: the minimum distance between centers of gratings for the shortest interferometers is $2L_{BG},$ and the difference in the cavity lengths of any two Fabry-Perot interferometers must exceed $2L_{BG}$ . 

Our future research work is in the following direction: wavelength-division multiplexing (WDM) can be implemented based on the low-finesse Fabry-Perot interferometers. The theoretical resolution is another direction. Technical applications are possible, for example: temperature, strain, humidity, force measurement, and oil detection. 

## 8. Conclusions

The quasi-distributed optical fiber sensor based on the low-finesse Fabry-Perot interferometer was studied theoretically and simulated numerically. Theory and simulation are in concordance. Our study considers quasi-distributed sensor properties, local sensor properties, signal processing, noise source, frequency-division multiplexing, and instrumentation. Our numerical results showed that all Fabry-Perot sensors have two resolutions: low resolution and high resolution. Low resolution is similar for all sensors, however, each Fabry-Perot sensor has its own high resolution. The thresholding value (from high resolution to low resolution) was defined in terms of low resolution and physical parameters. 

The quasi-distributed sensor has potential industrial applications, for example: structure monitoring, security system, humidity sensing, and level sensing. 

## Figures and Tables

**Table 1:** Quasi-distributed sensor parameters.

Sensor Number	Sensor Parameters	Signal Values
Low-finesse Fabry-Perot interferometer 1 (S1)	*L_FP1_* = 4 [mm]	${\Delta \lambda }_{BG}=3.22$ [nm] (Equation (3)) $\nu_{FP1}=4.95$ [Ciclos/nm] (Equation (4)) $\nu_{BG}=1.23$ [Ciclos/nm] (Equation (9))
	*L_BG_* = 0.5 [mm]	
	*n* = 1.46	
	$\lambda_{BG}=1532.5$ [nm]	
Low-finesse Fabry-Perot interferometer 2 (S2)	*L_FP2_* = 8 [mm]	${\Delta \lambda }_{BG}=3.22$ [nm] (Equation (3)) $\nu_{FP2}=9.91$ [Ciclos/nm] (Equation (4)) $\nu_{BG}=1.23$ [Ciclos/nm] (Equation (9))
	*L_BG_* = 0.5 [mm]	
	*n* = 1.46	
	$\lambda_{BG}=1532.5$ [nm]	
Low-finesse Fabry-Perot interferometer 3 (S3)	*L_FP3_* = 16 [mm]	${\Delta \lambda }_{BG}=3.22$ [nm] (Equation (3)) $\nu_{FP3}=19.82$ [Ciclos/nm] (Equation (4)) $\nu_{BG}=1.23$ [Ciclos/nm] (Equation (9))
	*L_BG_* = 0.5 [mm]	
	*n* = 1.46	
	$\lambda_{BG}=1532.5$ [nm]	

**Table 2:** Quasi-distributed sensor limits. (Δλ=10 pm)

Parameters	Value	Equation
$L_{FPmin}$	1 [mm]	Equation (16)
$L_{FPM}$	40.2 [mm]	Equation (20)
$L_{FPmin}\leq L_{FP}\leq L_{FPM}$	$1\leq L_{FP}\leq 40$ [mm]	Equation (21)
M	40	Equations (23) and (24)
$\nu_{max}$	102.47 [Ciclos/nm]	Equation (25)
$\nu_{s}$	204.95 [Ciclos/nm]	Equation (26)
